# Positive Effect of Gushukang on Type-H Vessel and Bone Formation

**DOI:** 10.3389/fcell.2020.00265

**Published:** 2020-05-21

**Authors:** Wantao Li, Xiaoqing Zhou, Tiejian Jiang, Hongbo He, Ting Wen

**Affiliations:** ^1^Department of Endocrinology, Endocrinology Research Center, Xiangya Hospital of Central South University, Changsha, China; ^2^Department of Orthopedic, Xiangya Hospital of Central South University, Changsha, China

**Keywords:** Gushukang, bone mass, bone formation, type-H vessel, HIF-1α

## Abstract

Gushukang (GSK) is a traditional herbal compound used in Chinese medicine for the treatment of osteoporosis. Numerous studies have been conducted to elucidate the effects of GSK, but the mechanisms underlying these effects remain unclear. In the present study, we cultured osteoblasts and osteoclasts with low and high doses of GSK, and also administered 3-month-old mice with 4 and 8 g/kg/day of GSK solution. Gushukang was found to promote osteoblast differentiation and inhibit osteoclast differentiation *in vitro*. *In vivo*, mice in the GSK treatment groups showed an increase in bone mass, as measured by micro-computed tomography (Micro-CT). Tartrate resistant acid phosphatase (TRAP) staining and osteocalcin (OCN) staining experiments revealed decreased bone resorption and increased bone formation in the GSK treatment groups. In addition, we found a novel effect of GSK—it could induce type-H vessel formation in mice. The underlying mechanisms of these actions were further explored at the molecular level to investigate whether these effects were due to an overexpression of the hypoxia inducible factor-1 (HIF-1α). Our findings indicate the utility of GSK as a therapeutic for the prevention of osteoporosis.

## Introduction

Primary osteoporosis is a bone disease characterized by decreased bone mass and bone density, resulting from estrogen deficiency and aging (Ng et al., 2015; Wang et al., 2015; Su et al., 2019; Rendina-Ruedy and Rosen, 2020). The risk of fractures is increased in primary osteoporosis, with the potential for serious disability and increased mortality in afflicted patients (Schett and Bozec, 2014; Nevius et al., 2015; Yang et al., 2019). The current treatment for osteoporosis comprises antiresorptive and bone-forming medicines (Cao et al., 2018), including estrogens (Hayashi et al., 2019), parathyroid hormone (Fan et al., 2017), bisphosphonates (Wei et al., 2016), and drugs, which blockade follicular stimulating hormone (Sponton and Kajimura, 2017). Most of these treatments concentrate on a single aspect of the disease, and thorough assessments of their efficacy and safety is still needed (Rauch et al., 2010). Thus, an exploration of new safer and more effective therapies is warranted.

Traditional Chinese herbal medicines have recently gained interest among medical researchers due to their low cost and limited side effects, and this renewed interest includes Gushukang (GSK), a traditional medicine made up of several traditional herbs, including *Herba Epimedii, Rehmannia glutinosa*, and *Rhizoma Drynariae* (Li et al., 2015; Xiao et al., 2020). Gushukang has already been approved for listing in the Chinese Pharmacopeia for the prevention and treatment of osteoporosis.

In clinical experiments, GSK–*Herba Epimedii*, an important GSK-containing formulation, has been reported to demonstrate positive effects on bone health (Indran et al., 2016). In a pharmacological study, GSK was demonstrated to increase bone density and promote the healing of bone fractures (Wang et al., 2007). Additionally, bone loss was prevented in hens fed with GSK, and an improvement in egg production was observed (Zhou et al., 2009), which suggests that the anti-osteoporosis effect of GSK is across species. In recent studies aimed at understanding the specific mechanisms underlying this action, Li et al. (2019) revealed that GSK modulates calcium homeostasis in OVX mice and that it has a favorable effect on bone formation (Li et al., 2019). In another study, this favorable effect was demonstrated to be related to the BMP-2/Smads signaling pathway (Chai et al., 2019). In summary, GSK is beneficial for bone mass development, although details of the underlying mechanisms remain unclear. Here, we reveal a new mechanism of GSK action that may pave the way for future osteoporosis treatments.

## Materials and Methods

### Experimental Animals

C57BL mice were purchased from the Experimental Animal Center of Central South University and raised in pathogen-free facilities, under a 12-h light/dark cycle as described previously (Li et al., 2018). The mice were provided with ample plastic bedding and clean water. All the experimental protocols were approved by the Subcommittee on Research and Animal Care (SRAC) of Central South University.

### Tissue Collection

Drugs were administered daily at 9:00–11:00 AM for 3 months as in the study by Kondegowda et al. (2015). Next, 24, 3-month-old mice were randomly divided into three groups: the control group, the low-dose group, and the high-dose GSK treatment group. Gushukang was dissolved in PBS and then administered. Mice in the low-dose group were administered 4 g/kg/day of GSK solution, whereas mice in the high-dose group were administered 8 g/kg/day of GSK solution intragastrically each day (Li et al., 2019). Three months later, the mice were killed by euthanasia. The femurs were collected and dissected free of soft tissue, fixed in 4% paraformaldehyde solution, and maintained in PBS until micro-computed tomography (Micro-CT) analysis, histomorphometry, and immunohistochemistry (Goldberg and Dixit, 2019).

### Osteoclast Differentiation

Bone marrow-derived macrophages (BMMs) were cultured as described by Schmitz et al. (2015). C57BL/6 mice aged 4 weeks were used to obtain BMMs. Cells from the bone marrow cavity were flushed with PBS and centrifuged at 1,000 rpm at 37°C. After centrifugation, the cells were cultured overnight with 100 ng/ml macrophage colony-stimulating factor (MCSF, 416-ML/CF, R&D, Minneapolis, MN, United States). Next, the unattached cells were gathered and further cultured for 48 h until sufficient BMMs were obtained. Gushukang serum was prepared as described previously (Zhao et al., 2019). Bone marrow-derived macrophages were cultured with 100 ng/ml macrophage colony-stimulating factor and 50 ng/ml receptor activator for nuclear factor-κB ligand (RANKL, 462-TR/CF, R&D, Minneapolis, MN, United States). Then, they were treated either with a low dose (20 μl/well) or a high dose (200 μl/well) of GSK serum. The osteoclasts were allowed to differentiate for 8 days, following which the effect of the GSK serum was evaluated by Tartrate resistant acid phosphatase (TRAP) staining. An osteoclast was defined as a positively stained cell with more than three nuclei (Berger et al., 2019).

### Osteoblast Differentiation

Osteoblasts were cultured as described in a previous study (Yu et al., 2019). The femurs were cut into small pieces and treated with 1 mg/ml collagenase solution, containing collagenase type I and collagenase type II at 1:3 ratio (Worthington, Newark, NJ, United States). Then, bone marrow-derived mesenchymal stem cells (BMSCs) were collected and cultured in Dulbecco’s Modified Eagle’s Medium (Invitrogen, Carlsbad, CA, United States), to which 10% fetal bovine serum (Invitrogen, New Zealand) and 100 U/ml penicillin and streptomycin (Invitrogen) were added. The cells were cultured with 50 μM ascorbic acid, 50 mM β-glycerophosphate, 50 nM dexamethasone (Sigma, St. Louis, MO, United States), and GSK serum (a low dose of 200 μl/well or a high dose of 2 ml/well) for 14 days. Next, the cells were fixed with 4% paraformaldehyde solution and then stained with 1% Alizarin red S (pH 4.2, Sigma-Aldrich, GmbH, Munich, Germany) for 10 min. After washing with PBS three times, four visual fields were randomly selected and from each slide and analyzed. The volume of mineralized bone nodules was quantified by image J software.

### Immunohistochemistry Staining

Immunohistochemical staining was performed as described in previous studies (Albrecht et al., 2011; Chen et al., 2020). Fresh femurs were obtained and fixed in 4% paraformaldehyde solution at 4°C for 24 h, and then they were placed in 10% ethylene diamine tetraacetic acid solution (pH 7.4) and decalcified for 21 days. Next, the bones were embedded in paraffin. Paraffin wax blocks were sliced into longitudinally oriented bone sections with a thickness of 4 μm. Osteocalcin staining was performed to determine the number of osteoblasts and study their surface characteristics. Tartrate resistant acid phosphatase staining was performed to determine the number of osteoclasts and study their surface characteristics. Both the staining kits were purchased from Sigma Company (St. Louis, MO, United States). The distal metaphysic area of each femur was chosen for counting the number of positive-stained cells in four random visual fields from five consecutive sections of each mouse. Then, they were normalized to the number per millimeter in the adjacent bone surface (N mm^–1^).

### Immunofluorescence Staining

Immunofluorescence staining was conducted as described in previous studies (Rached et al., 2010; Yang et al., 2017). Fresh bone tissues were collected and fixed immediately in 4% paraformaldehyde solution at 4°C for 4 h. Subsequently, the bone tissues were placed in 0.5 M ethylene diamine tetraacetic acid solution (pH 7.4) for decalcification at 4°C for 48 h, followed by dehydration with 20% sucrose and 2% polyvinylpyrrolidone (PVP) solution for 1 day until they descended to the bottom. Next, 8% gelatin (porcine; Sigma, G2500), 20% sucrose, and 2% PVP (Sigma, PVP360) were mixed and dissolved with PBS at 60°C. Finally, the tissues were embedded as described above. Forty-micrometer-thick, longitudinally oriented bone sections were sliced and stained with primary antibodies of mouse CD31 (Abcam, ab28364, 1:100) and endomucin (Santa Cruz, V.7C7, 1:50) overnight at 4°C. Next, the sections were incubated with secondary antibodies (Jackson ImmunoResearch, 415-605-166, 1:500; 315-545-003, 1:500) at 37°C for 1 h, away from light. Polyclonal goat IgG (R&D Systems, AB-108-C) and monoclonal rat IgG2A (R&D Systems, 54447) were used as negative controls. The specimens were observed using a confocal microscope (FLUOVIEW FV300, Olympus).

### Micro-Computed Tomography Analysis

Bone structure was measured by high-resolution micro-CT as described in Mills et al.’s (2016) study. The femurs were scanned at 0.5 mm under the growth plate with a voltage of 70 kV and a current of 154 mA. The following software packages were used for the three-dimensional analysis of bones: image reconstruction software (NRecon v1.6), three-dimensional model visualization software (mCTVol v2.0), and data analysis software (CTAn v1.9). Bone parameters were expressed as trabecular bone volume per tissue volume (Tb. BV/TV), trabecular number (Tb. N), trabecular thickness (Tb. Th), and trabecular separation (Tb. Sp).

### Western Blotting

Protein levels were measured as described in Stier et al.’s (2018) study. The femurs were ground in a mortar after adding liquid nitrogen. The powders obtained from each bone sample were treated with a 500 μl RIPA buffer for 20 min. After centrifugation, protein lysates were obtained, which were then transferred to SDS at a ratio of 4:1 and boiled at 100°C for 10 min. The lysates were separated by 10% SDS-PAGE and blotted onto a PVDF membrane. After being blocked with milk for 60 min, the membrane was treated with primary antibodies against HIF-1α (Cell Signaling Technology, Danvers, MA, United States) overnight at 4°C and then incubated with goat antirabbit immunoglobulin (1:2,000; Santa Cruz Biotechnology) for 1 h at 37°C. Finally the bands were developed using ELC reagents.

### QT-PCR

The femur and tibia were collected from each mice. mRNA levels were determined as described in Walter et al.’s (2014) study. Total RNA was extracted from bone tissues by using the TRIzol reagent (Invitrogen, Carlsbad, CA, United States); this was followed by complementary DNA (cDNA) synthesis using a cDNA Kit (Pharmacia, Piscataway, NJ, United States). QT-PCR was carried out by using a KAPA SYBR FAST qPCR Kit (KAPA Biosystems, Wilmington, MA, United States) and an ABI PRISM^®^ 7900HT System (Applied Biosystems, Foster City, CA, United States). The primer sequence of HIF-1a was shown as follows: forward (5′–3′) TCTGGAAGGTATGTGGCATT, reverse (5′–3′) AGGGTGGGCAGAACATTTAT. mRNA expression was determined by using the 2^–Δ Δ CT^ method (Ouyang et al., 2018).

### Statistical Analysis

All data are shown as mean ± SD for each group. One-way ANOVA was used for analyzing differences among the three groups (groups of control, low-dose, and high-dose GSK treatment) with PRISM, version 7.0 (GraphPad), as described in Wei et al.’s (2014) study. All experiments were conducted more than three times. Differences were considered to be significant only when *P* < 0.05.

## Results

### GSK Promoted Bone Formation in Mice

**FIGURE 1:**
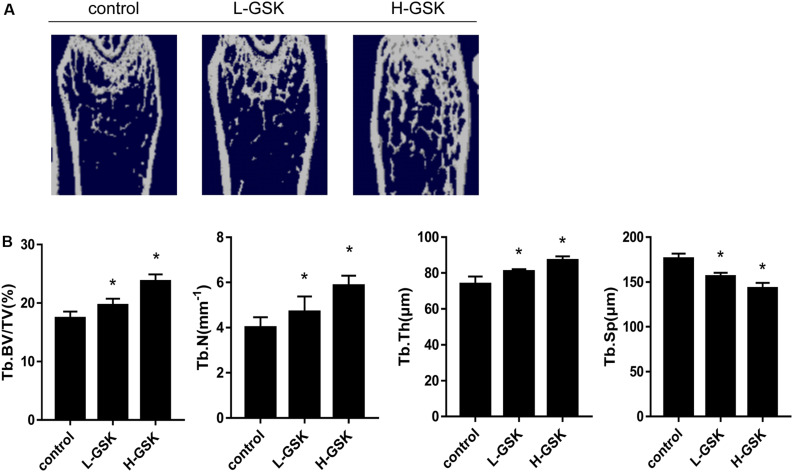
GSK prevented bone loss in mice. **(A)** Representative μCT images of femurs collected from mice treated with low-dose (4 g/kg/day) and high-dose (8 g/kg/day) GSK or control. **(B)** Quantitation of Tb.BV/TV (trabecular bone volume per tissue volume); Tb.N (trabecular number); Tb.Th (trabecular thickness); and Tb.Sp (trabecular separation). Values were expressed as mean ± SD.*P < 0.05; all the assays were repeated more than three times.

Micro-computed tomography is a commonly used method for the measurement of bone mass density (Hirose et al., 2014). We found that the GSK-treated mice had higher values of bone mineral density (BMD) than the control group mice (Figure 1A). Moreover, mice treated with a low dose of GSK had a lower BMD than mice treated with a high dose of GSK. Important 3D outcomes of micro-CT analysis including BV/TV, Tb.N, and Tb.Th were higher in the GSK treatment groups than in the control group (Figure 1B; *P* < 0.05). Moreover, these values were lower in the low-dose GSK treatment group than in the high-dose GSK group. In contrast, trabecular separation (Tb.Sp) was lower in the GSK treatment groups (Figure 1B; *P* < 0.05). These results indicate that GSK treatment had a positive effect on bone formation.

### GSK Increased Osteoblastogenesis *in vivo* and *in vitro*

**FIGURE 2:**
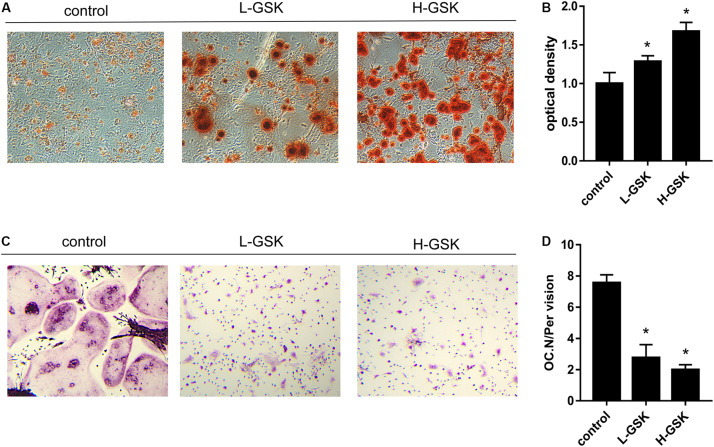
GSK increases osteogenic differentiation and decreases osteoclast differentiation *in vitro*. **(A)** BMSCs were gathered from C57BL/6 mice (4 weeks) and cultured with GSK serum. Alizarin red staining was carried out at day 14 to assess osteogenic differentiation. Scale bar = 100 μm. **(B)** The Alizarin red staining optical density was quantified by ImageJ. *P < 0.05; the groups of GSK versus control. **(C)** BMMs were obtained from 4 weeks C57BL/6 mice and treated with M-CSF (100 ng/ml) and RANKL (50 ng/ml) (control), M-CSF, and RANKL added GSK serum. Osteoclast differentiation was evaluated at day 8 by TRAP staining. Scale bar = 100 μm. **(D)** The number of osteoclasts was quantified. Values were expressed as mean ± SD. *P < 0.05; all the assays were repeated more than three times.

**FIGURE 3:**
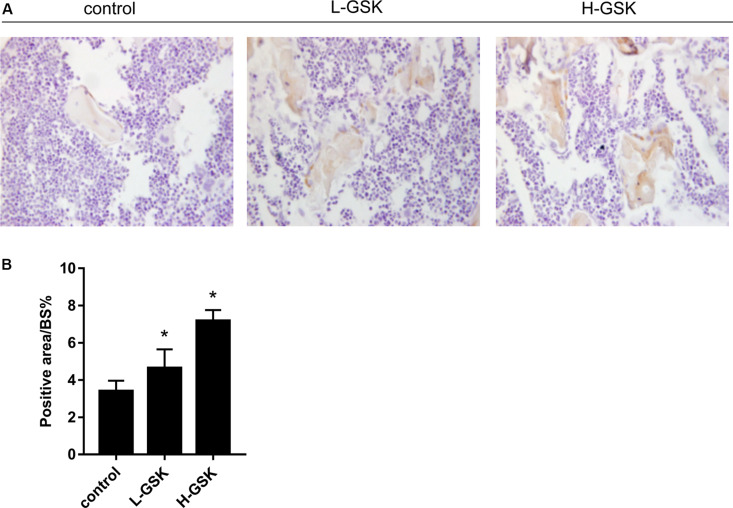
GSK promoted the expressions of OCN in mice. **(A,B)** The protein expression of osteocalcin (OCN) of femurs gathered from mice treated with low-dose and high-dose GSK or control (saline) was determined by immunohistological staining. Scale bar = 100 μm. Values were expressed as mean ± SD. *P < 0.05; all the assays were repeated more than three times.

To determine whether GSK increased bone mass by affecting the process of osteoblastogenesis, we cultured osteoblasts *in vitro*. Alizarin red staining demonstrated that formation of mineralized bone nodules was increased in cells treated with GSK compared to that in the control cells, and that the formation of additional mineralized bone nodules was dose dependent (Figure 2A). The optical density of the mineralized bone nodules was quantified as illustrated in Figure 2B (*P* < 0.05). The results obtained *in vivo* following GSK treatment are consistent with these *in vitro* results. Immunohistochemistry staining showed that the femurs from GSK-treated mice expressed a higher ratio of osteocalcin (OCN)-positive area surface to bone area than femurs from control mice. Moreover, the high-dose GSK group showed a higher ratio than the low-dose GSK group (Figures 3A,B; *P* < 0.05). As OCN is an important osteogenic differentiation biomarker (Li et al., 2009), the results indicate that GSK could increase bone mass partly by inducing osteoblast differentiation.

### GSK Inhibited Osteoclastogenesis *in vivo* and *in vitro*

**FIGURE 4:**
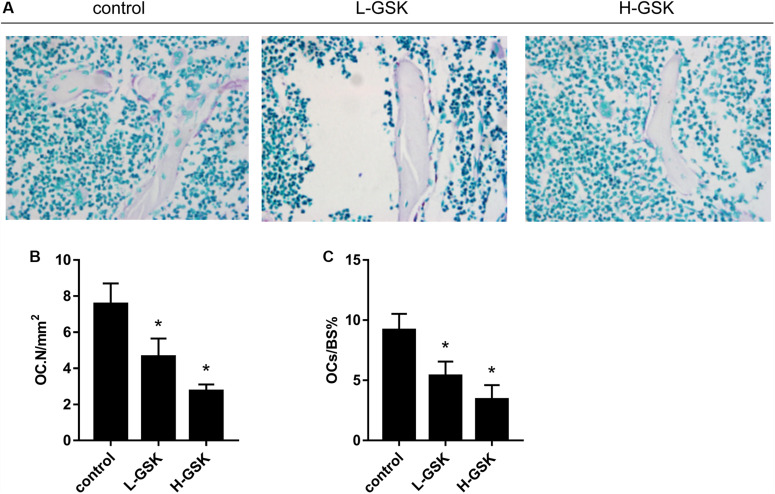
GSK decreases the number of osteoclasts in mice. **(A)** TRAP staining was performed on femurs collected from the group of low-dose and high-dose GSK or control (saline). Scale bar = 100 μm. **(B)** Osteoclast-covered surface over bone surface (OCs/BS%) of each group was quantified. **(C)** Osteoclast number (OC.N). Values were expressed as mean ± standard deviation (SD). *P < 0.05; all the assays were repeated more than three times.

We next investigated the influence of GSK on the process of osteoclastogenesis. Osteoclasts were induced from BMMs and quantified as shown in Figure 2D (*P* < 0.05). We observed that the number of osteoclasts was significantly suppressed in the GSK treatment groups, and that this inhibitory capacity increased with increasing doses of GSK (Figure 2C). This observation was subsequently confirmed *in vivo*, since TRAP-positive osteoclasts were significantly decreased in GSK treatment groups, with high-dose GSK treatment groups showing fewer positive cells than the low-dose groups (Figure 4A). When the ratio of OCs to%BS and the absolute number of OCs were quantified, the GSK treatment groups showed fewer osteoclasts per area than the control groups (Figures 4B,C; *P* < 0.05). Thus, our findings provide evidence that GSK treatment also increases bone mass by inhibiting osteoclastogenesis.

### GSK Accelerated Type-H Vessels Formation in Mice

**FIGURE 5:**
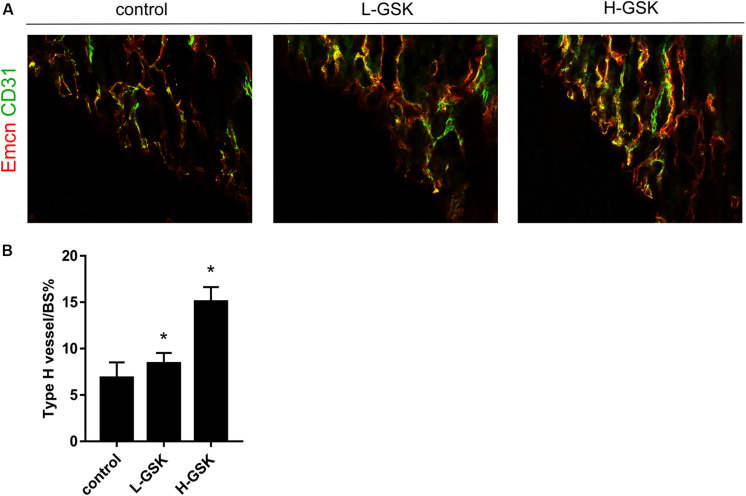
GSK accelerated CD31hiEmcnhi vessels formation in mice. **(A)** Representative images of CD31 (green), Emcn (red) of femurs from the group of low-dose and high-dose GSK or control (saline) by immunofluorescence staining. Scale bar = 100 μm. **(B)** Type-H vessel surface was quantified. Values were expressed as mean ± SD. *P < 0.05; all the assays were repeated more than three times.

Type-H vessels are associated with the differentiation of perivascular osteoprogenitors and bone formation (Weber et al., 2015). The type-H vessels are characterized as CD31 positive and endomucin positive (Morrison et al., 2014). The GSK treatment groups had more type-H vessels, as revealed by the number of CD31^hi^Emcn^hi^ endothelial cells, than the control group, and the formation of type-H vessels was higher in the high-dose GSK treatment group than in the low-dose group (Figure 5A). The ratio of type-H vessels to BS% was quantified as shown in Figure 5B (*P* < 0.05).

### GSK Promoted the Activation of HIF-1a

**FIGURE 6:**
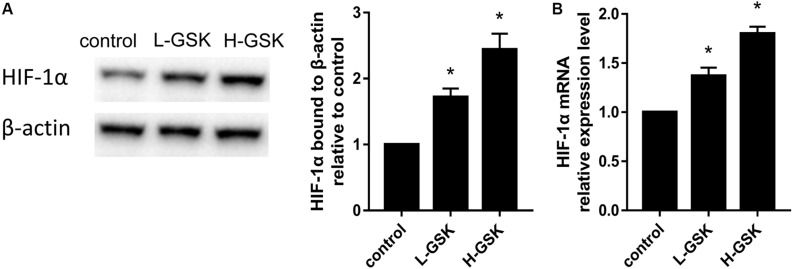
GSK induced overexpression of HIF-1a. **(A)** Representative images of WB and quantification of the protein expression level of HIF-1a. **(B)** Quantification of the mRNA expression level of HIF-1a. Values were expressed as mean ± SD. *P < 0.05; all the assays were repeated more than three times.

The hypoxia inducible factor-1α (HIF-1α) is an important transcription factor that is activated under hypoxic conditions (Dai et al., 2019) and during the process of angiogenesis (Zhang et al., 2010). Hypoxia inducible factor-1α expression levels were increased in the GSK treatment groups, and HIF-1α expression was dependent on the GSK dose (Figure 6A; *P* < 0.05). These results suggest that GSK was able to upregulate the expression of HIF-1α protein. Moreover, when qRT-PCR was performed to assess the mRNA expression levels of *HIF-1*α, the GSK treatment groups showed higher levels of *HIF-1*α mRNA expression than the control group, and the high-dose group showed higher mRNA levels than the low-dose group (Figure 6B; *P* < 0.05). Thus, our data suggest that GSK can induce *HIF-1*α activation.

## Discussion

Gushukang, a traditional Chinese herbal medicine, has been used for the treatment of osteoporosis for many years, and the significant effects of this compound have been certified by clinical practices and approved by patients. Li et al. (2001) selected 197 male and female osteoporosis patients, and GSK was either administered (GSK treatment group) or not administered (control group) to patients randomly assigned to these groups (Li et al., 2001). Six months after GSK treatment, while BMD had decreased in the control group, the BMD decrease was slowed, and BMD even increased in some cases, in the GSK-treated group.

Here, we explored the underlying mechanism of GSK action and found that cells in the GSK treatment group demonstrated enhanced osteoblastogenesis during the period of osteoblast differentiation from BMSCs. In addition, the number of osteoclasts in the GSK treatment groups was lower than in the control groups, suggesting that the osteoclastogenesis ability of BMMs was inhibited by GSK. These results were found to be consistent with previous research (Wang et al., 2018). Thus, we conclude that GSK increases osteoblastogenesis and suppresses osteoclastogenesis *in vitro*.

To further understand the role of GSK *in vivo*, we evaluated bone mass and bone biochemical markers in mice. Using micro-CT, increased cortical and cancellous bone mass in the GSK treatment group could be inferred from the higher BV/TV, Tb.N, and Tb.Th and the lower Tb.Sp values observed in this group than in the control group. These results suggest that bone anabolism is greater than bone catabolism after GSK treatment, confirming that GSK enhances bone density and bone mass. Tartrate-resistant acid phosphatase, produced by osteoclasts, and OCN, secreted by osteoblasts (Booth et al., 2016; Calvier et al., 2017), levels reflect the activity of osteoclasts and osteoblasts as well as the status of bone resorption and bone formation (Long et al., 2014; Yu et al., 2015; Huang et al., 2020). In our study, TRAP and OCN staining results suggest that GSK treatment increased bone mass by activating osteoblasts and suppressing osteoclasts. Thus, bone formation was promoted and bone resorption was inhibited. This result was consistent with the results we obtained in cells, verifying the osteogenesis activity of GSK *in vivo*.

Type-H vessels are a special subtype of vessel in bone characterized by high levels of endomucin and CD31 expression (Diebold et al., 2015). Type-H vessels have recently been shown to possess the ability to induce angiogenesis and bone formation (Peng et al., 2020). Our results show that GSK-treated mice have more CD31^hi^Emcn^hi^ vessels than control mice, which suggests that GSK may have increased the abundance of type-H vessels. These results are consistent with the notion that angiogenesis and bone formation are coupled. This is the first study to report a role for GSK in angiogenesis and specifically in the formation of type-H vessels. This newfound action of GSK may find additional application in other diseases.

The mechanism underlying the effect of GSK on type-H vessels and coupling with angiogenesis were further investigated. Previous studies have shown that GSK upregulated calcium-binding protein-28k and the vitamin D receptor (Li et al., 2019), and also Osteirx and Runx2 (Wang et al., 2018). In this study, we reveal that HIF-1α, a factor produced under hypoxic conditions (Baik et al., 2019), may also play a regulatory role, with its overexpression contributing to angiogenesis and osteogenesis (Stegen et al., 2016; Hulea et al., 2018). The results of our western blot analyses show that bones from the GSK treatment group expressed higher HIF-1α protein levels and that this increase was dependent on the GSK dose. Similarly, *HIF-1*α mRNA levels measured by qRT-PCR were higher in the GSK treatment group than in the control group. These data suggest that GSK treatment may induce formation of type-H vessels and bone formation by enhancing the expression of *HIF-1*α.

## Conclusion

In conclusion, GSK can increase bone mass by promoting bone formation and the formation of the type-H vessels, and by inhibiting bone resorption. These functions may be related to HIF-1α activity. The results of our study may advance new therapeutic treatments for the prevention of osteoporosis.

## Data Availability Statement

The datasets generated for this study are available on request to the corresponding author.

## Ethics Statement

The animal study was reviewed and approved by the Subcommittee on Research and Animal Care (SRAC) of Central South University.

## Author Contributions

TW designed and guided the experiment. WL and XZ carried out the experiment. TJ analyzed the data. WL and HH wrote the manuscript. All authors contributed to manuscript revision and read and approved the submitted version.

## Conflict of Interest

The authors declare that the research was conducted in the absence of any commercial or financial relationships that could be construed as a potential conflict of interest.
